# Editorial: Viruses and Epitranscriptomes: Regulation of Infection and Antiviral Response

**DOI:** 10.3389/fcell.2022.917894

**Published:** 2022-05-09

**Authors:** Ana Raquel Soares, Marjolein Kikkert, Stefanie Kellner-Kaiser, Daniela Ribeiro

**Affiliations:** ^1^ Department of Medical Sciences, Institute of Biomedicine—IBiMED, University of Aveiro, Aveiro, Portugal; ^2^ Molecular Virology Laboratory, Leiden University Medical Center, Leiden University Center of Infectious Diseases (LU-CID), Leiden, Netherlands; ^3^ Institute of Pharmaceutical Chemistry, Goethe-University Frankfurt, Frankfurt, Germany

**Keywords:** virus, RNA modifications, epitranscriptome, antiviral response, RNA

It is widely known that viruses rely on the host cell machinery to translate their own genomes (Stern-Ginossar et al., 2019; Nunes et al., 2020). This depends on highly intricate mechanisms that are still far from being totally understood.

In recent years, an increasing number of reports have emerged demonstrating that viruses interact with the host RNA modification machinery, highlighting the importance of epitranscriptomic marks for viral infection (Pereira-Montecinos et al., 2017). Both host and viral RNA molecules are highly post-transcriptionally modified. These modifications are important modulators of gene expression and, to date, more than 170 different modifications, regulated by different RNA modification proteins (RMPs), have been identified (Wiener and Schwartz, 2021).

Distinctive epitranscriptome marks have been recently found on total RNA extracted from cells infected with different RNA viruses, suggesting that RNA post-transcriptional modifications may be important for viral propagation (McIntyre et al., 2018). The most common RNA modification identified both in humans and in viral transcripts is the addition of a methyl group to the N6 position of adenosine (m6A). This RNA modification seems to influence viruses’ life cycle and, for that reason, it is considered a potential cellular antiviral target (Tan and Gao, 2018; Dang et al., 2019; Baquero-Perez et al., 2021). Nevertheless, the impact of the epitranscriptome in infection and viral genome translation is still largely underestimated. This research topic was set to increase the knowledge in this field and promote scientific debate. It attracted different researchers from around the globe and resulted in 4 publications.

Interestingly, the majority of the submissions were focused on the severe acute respiratory syndrome coronavirus 2 (SARS-CoV2) and highlighted the increasing relevance of the epitranscriptome in the context of this viral infection.

Izadpanah and colleagues discussed the increasing evidence of the effects of several RNA modifications, namely m6A, pseudouridine (Ψ), and 2′-O-methylation (Nm) on SARS-CoV2 replication (Izadpanah et al.). They have particularly addressed the role and expression levels of the different RMPs in the lungs of SARS-CoV-2 infected patients and their impact on RNA modification levels were discussed to enable novel therapeutically strategies targeting the epitranscriptome.

Tacking advantage of high-throughput sequencing technologies, Peña and colleagues characterized the small and large RNAs from different SARS-CoV-2 viral isolates (Peña et al.). These proof-of-concept studies found a selective enrichment of specific host transfer RNAs (tRNAs), tRNA fragments and signal recognition particles. Additionally, tRNA modifications diverged in infected and non-infected cells, highlighting that host tRNA modifications are also affected by viral infection. Due to the important role of tRNAs in translation, it is possible that altered tRNA modification levels will condition tRNA availability and stability, which ultimately affects translation levels. In addition to these findings, the authors were also able to identify additional modifications in the SARS-CoV2 genomic RNA.

Figueroa and colleagues have specifically addressed the m6A modification and studied its impact on the human respiratory syncytial virus (HRSV) infection (Figueroa et al.). They have particularly analysed the importance of m6A writers, erasers and readers on HRSV genomic RNA accumulation and inclusion bodies assembly during viral replication. Their results suggest that m6A, through the YTHDF1-3 reader, induces a decrease in intracellular levels of viral genomic RNA and alters the dynamics of inclusion body assembly, consequently affecting viral replication. These data clearly illustrate the importance of the interplay between RNA modifications and inclusion body assembly in the context of viral infections.

In a comprehensive review, Lu and colleagues explored the relationship between viruses and extracellular vesicles (Yang et al.). Viral infections have been shown to modulate the number, composition and function of extracellular vesicles to increase propagation and pathogenicity. On the other hand, non-coding RNA (ncRNA) molecules are often targeted by viruses and have been identified as the main functional cargo of virus-related extracellular vesicles. The authors hence present and discuss the recent research progress concerning virus-regulated extracellular vesicles. Furthermore, due to the similarity between extracellular vesicles and some virus particles in terms of size and density, isolation of pure extracellular vesicles from infected cells poses important challenges. The authors address this important topic and present an informative discussion on the isolation strategies applied to virus-infected samples. In the future it will be interesting to evaluate modification levels in the ncRNAs found in the extracellular vesicles.

This Research Topic brought together different perspectives on the role of the epitranscriptome in the context of viral infections. This exciting convergence of fields sheds a light on the relevance of viral epitranscriptomics, host epitranscriptome alterations in response to viral infection, contribution of epitranscriptomic marks to viral replication, and the relevance of the epitranscriptome to other regulatory mechanisms. It is expected that an increasing number of studies focused on viral- and host-mediated epitranscriptome modulation and on the identification of the factors that regulate this phenomenon will be available in the next few years. These studies will be critical to understand gene regulation during viral infection, and likely identify the epitranscriptome as a druggable target.
